# Subcutaneous and Periorbital Emphysema Following a Dental Procedure

**DOI:** 10.7759/cureus.83089

**Published:** 2025-04-27

**Authors:** Takafumi Obara, Tsuyoshi Nojima, Kazuki Nakatsuji, Takashi Hongo, Tetsuya Yumoto, Atsunori Nakao

**Affiliations:** 1 Department of Emergency, Critical Care and Disaster Medicine, Faculty of Medicine, Dentistry and Pharmaceutical Sciences Okayama University, Okayama, JPN; 2 Department of Oral and Maxillofacial Reconstructive Surgery, Okayama University Hospital, Okayama, JPN

**Keywords:** air pressure, antibiotic prophylaxis, dental procedures, operative, subcutaneous emphysema

## Abstract

Subcutaneous emphysema following dental procedures is rare. We present the case of a young, healthy woman who was transferred from a dental clinic to our emergency department due to sudden swelling of the left orbit immediately after a dental procedure involving the use of the dental air and water syringe. The diagnosis of subcutaneous facial emphysema was made based on the patient's history, physical examination, and computed tomography imaging. The patient received prophylactic amoxicillin, and the lesion resolved completely in one week. Prompt clinical suspicion and a thorough evaluation of the signs and symptoms, including a detailed clinical history, are crucial for diagnosing subcutaneous emphysema following a dental procedure.

## Introduction

Subcutaneous emphysema in the head, neck, and thorax is characterized by swelling caused by the introduction of air into connective tissue spaces. This condition is mostly associated with trauma, gas-forming infections, and surgical procedures [1,2]. However, it is a rare complication of dental procedures including crown preparation, implant procedures, endodontic treatments, and restorative work [3]. Several case reports have described air entering the facial tissue spaces in association with dental procedures, typically owing to the use of high-speed dental handpieces with functioning air sprays during the sectioning of impacted teeth [1-8].

Although exact incidence rates are not well documented, some studies have characterized subcutaneous emphysema as an uncommon complication in dental practice [9]. The severity of reported cases varies widely, ranging from mild, self-limiting swelling to serious complications requiring intervention. In most instances, symptoms resolve spontaneously within a few days to a week without long-term sequelae [9,10]. However, rare but severe outcomes such as pneumomediastinum, tension pneumothorax, and air embolism have also been described [7,11]. Among physicians, subcutaneous emphysema following dental procedures is not widely recognized.

In this report, we present a case of subcutaneous periorbital emphysema in a young woman who underwent dental procedures. This case serves to raise awareness among clinicians, outline differential diagnoses, indicate the necessary diagnostic procedures, and recommend optimal management strategies to minimize unnecessary tests and reduce hospitalization costs.

## Case presentation

A healthy 21-year-old woman presented to our emergency department with acute swelling beneath her left eye following a dental procedure. The patient had no history of smoking, drug allergy, underlying diseases, or previous surgical history. According to her dentist, the palatal root fractured during suture removal from the maxillary left first premolar. The tooth was extracted using air pressure, but the procedure was halted due to swelling in the left cheek.

Although the exact mechanism of subcutaneous emphysema remains unclear, it is presumed that air was inadvertently introduced into the soft tissue spaces, during either the air-driven extraction or suture removal. This suspicion is based on the temporal association between instrument use and the onset of facial swelling. A local anesthetic was administered. According to the medical records, the patient had previously tolerated similar anesthetics without adverse reactions. The dentist briefly applied manual compression without improvement, prompting the patient to be transferred to our department.

On arrival, the patient showed no signs of respiratory distress. Her vital signs were within normal limits, including a blood pressure of 141/87 mmHg, a heart rate of 74 beats per minute, a respiratory rate of 16 breaths per minute, an oxygen saturation at a normal level, and a body temperature of 36.8°C. The patient was alert and responsive. She had no complaints of sore throat, visual disturbance, or diplopia. Physical examination revealed no skin swelling, erythema, warmth, purpura, or telangiectasia. There was palpable facial swelling, indicating subcutaneous emphysema, but the patient did not experience any pain, hoarseness, or limitation in mouth opening. No proptosis or restricted eye movement was observed. The heart and lung findings showed no abnormalities.

**Figure 1:**
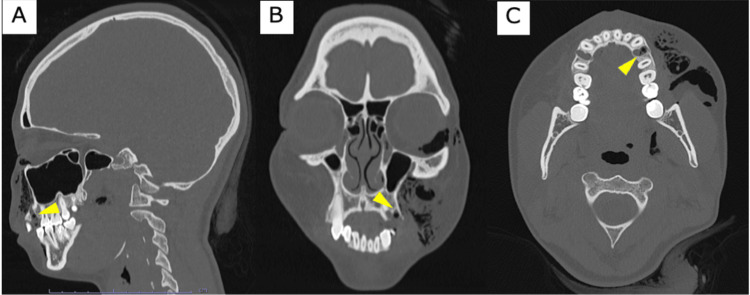
Facial emphysema Computed tomography of the neck and head at the time of emergency department presentation: (A) sagittal, (B) coronal, and (C) axial views. Radiolucent air pockets (dark areas) are visible in the subcutaneous tissues of the left midface, extending into the periorbital region. The likely point of entry is the extraction site of the left maxillary first premolar (indicated by the yellow triangle).

Computed tomography (CT) of the neck and head revealed extensive facial subcutaneous emphysema extending into the right orbital region, confirming the diagnosis of facial emphysema secondary to the dental procedure (Figure 1).

**Figure 2:**
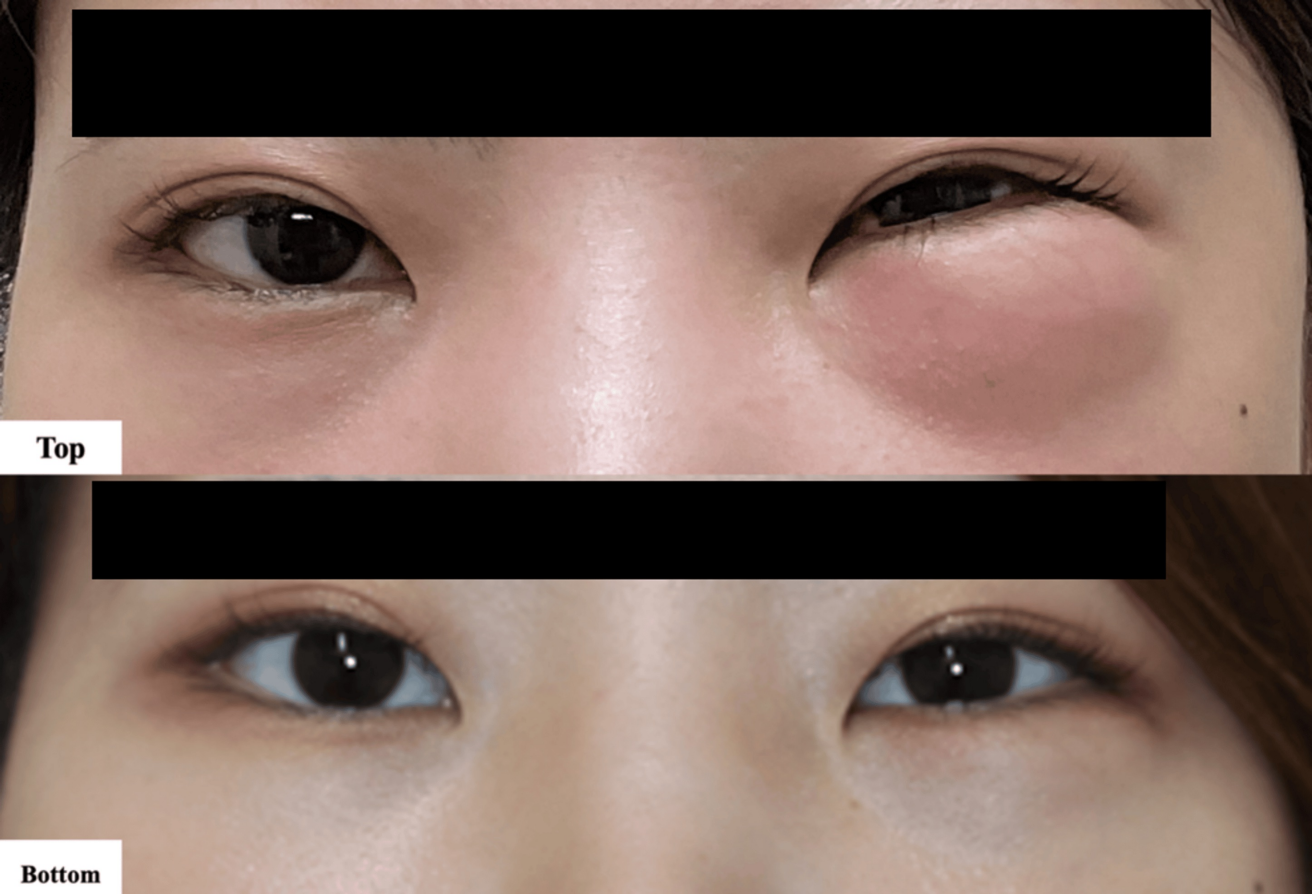
Photographs of periocular region (Top) At the time of emergency department visits. (Bottom) At the follow-up visit one week later.

Prophylactic antibiotics (amoxicillin 250 mg three times daily) were administered for seven days to prevent infection caused by oral bacteria. After two days, there was a significant reduction in edema and pain on palpation, with nearly imperceptible crepitus. At a follow-up appointment one week later, complete resolution of the facial edema was noted, with no change in visual assessment. No additional investigations were required as the patient recovered completely (Figure 2).

## Discussion

A previous report described the occurrence of subcutaneous emphysema in the left periorbital area of a young female patient following a dental procedure. This case report highlights a rare complication associated with dental procedures. Instruments such as handpieces and air/water syringes, which deliver air or water at high pressure, can forcefully introduce air into surrounding tissues, leading to subcutaneous emphysema.

Diagnosing this complication can be straightforward if there is a history of using an air-driven, high-pressure device. When examining the swollen area, healthcare providers may feel a sensation resembling "crackling" or "bubbles popping", which is medically referred to as crepitus. Radiolucent air layers may be visible on plain radiographs of the neck, particularly in larger or more advanced cases. However, plain radiographs often fail to detect small or localized air collections, making them less reliable for definitive diagnosis. In contrast, CT provides superior sensitivity and spatial resolution, enabling the precise identification of the location and extent of subcutaneous emphysema.

Although CT is the imaging modality of choice in most cases, X-ray imaging may still be useful in initial evaluations, particularly in emergency settings or when CT is unavailable. It may help detect large air collections and support a preliminary diagnosis; however, a negative X-ray does not rule out subcutaneous emphysema. Therefore, if clinical suspicion remains high, CT should be performed regardless of radiographic findings [12].

Multiple potential diagnoses should be evaluated when a patient experiences abrupt swelling in the head and neck area during or immediately after a dental procedure. These include anaphylaxis, angioedema, hematoma, and infection. Anaphylaxis was considered unlikely based on the patient's clinical history and physical examination. There were no signs of urticaria, hypotension, respiratory distress, or airway compromise at any time. Additionally, the local anesthetic and hemostatic agents used were reportedly identical to those previously administered without adverse reactions. While prior tolerance does not definitively exclude anaphylaxis, the overall clinical presentation was inconsistent with a systemic immunologic response. True anaphylaxis typically presents as more extensive bilateral swelling and potential cardiorespiratory issues. For angioedema, swelling typically appears more widespread and fluid-filled. Hematoma, on the other hand, presents as a mass due to the accumulation of blood from the submucosal hemorrhage. Both conditions were deemed unlikely given the sequence of events.

Most cases of subcutaneous facial emphysema are benign and self-limiting. A systematic review by Jones et al. reported that the majority of cases resolved within 2-10 days without the need for invasive intervention [9]. The clinical course is further supported by findings from other case reports [10]. However, because severe and potentially life-threatening complications can arise, careful observation is crucial to monitor for any signs of airway compromise. Serious complications may occur if air enters the parapharyngeal or retropharyngeal spaces, potentially leading to pneumomediastinum or pneumothorax [11]. Other reported complications include tension pneumothorax, air emboli, and blindness [7]. In particular, if significant changes such as proptosis, vision loss, or increased intraocular or intraorbital pressure are observed, orbital decompression is necessary. Successful techniques for relieving air mass pressure include direct methods, such as using a syringe with an attached needle to decompress the area, as well as indirect approaches, such as lateral canthotomy and cantholysis procedures [13]. These emergency procedures are typically indicated in cases of orbital compartment syndrome, where rapid decompression is required to preserve vision.

This case is notable in that the patient was a young, otherwise healthy woman, with emphysema extending from the facial region into the periorbital area. Although subcutaneous emphysema following dental procedures has been reported across various age groups, most published cases involve middle-aged adults [9]. These features make this case relatively uncommon and highlight the importance of recognizing atypical presentations of subcutaneous emphysema.

Although there is a potential for microorganisms from the oral flora to migrate into air-invaded spaces, the use of broad-spectrum antibiotics is not well established due to a lack of clear evidence supporting their efficacy in this context. In some cases, adjunctive therapies such as 100% oxygen or corticosteroids have been used to promote air resorption and reduce inflammation, although their routine use remains controversial [14]. Given this and the absence of signs indicating an infectious etiology, our patient was administered prophylactic antibiotics at her request (amoxicillin 750 mg/day). Subsequently, no infectious complications were observed, and the patient recovered completely.

## Conclusions

Although uncommon, subcutaneous facial emphysema can occur because of standard dental restoration procedures. It is crucial for healthcare professionals to have a comprehensive understanding of this potential complication to ensure an accurate diagnosis and appropriate treatment.
